# Primary leiomyosarcoma of the male urethra: a case report

**DOI:** 10.1186/1757-1626-2-207

**Published:** 2009-11-18

**Authors:** Youness Ahallal, Mohammed Fadl Tazi, Abdelhak Khallouk, Elmehdi Tazi, Amal Benlemlih, Mohammed Jamal El Fassi, Moulay Hassan Farih

**Affiliations:** 1 Department of Urology, Teaching Hospita Hassan II, Fez, 30000, Morocco; 2 Department of Oncology, National Institute of Oncology, Teaching Hospital Ibn Sina, 1000, Rabat, Morocco; 3 Department of Histopathology, Teaching Hospital Hassan II, Fez, 30000, Morocco

## Abstract

**Background:**

Cancers of the male urethra constitute less than 1% of all malignant urological tumours, and the occurrence of sarcomas in the urethra is even less frequent. To our knowledge, only one case has been previously described in the English literature.

**Case presentation:**

We report the clinical features, histology, imaging and treatment of urethral leiomyosarcoma in a male patient.

**Conclusion:**

The occurrence of sarcoma in the urethra is most unusual, and its appearance as a primary growth in the male urethra is not recorded in the English literature. In conclusion, this case highlights a rare type of primary male urethral malignancy that features a poor prognosis.

## Introduction

Primary sarcoma of the urethra in the male patient is extremely rare. Malignant neoplasms of the urethra are typically squamous cell carcinoma. Leiomyosarcoma is a very rare neoplasm of the urinary system with less than 20 cases being reported in the literature up to date.

We report below the case of a male patient with ureteral leiomyosarcoma.

## Case presentation

**Figure 1:**
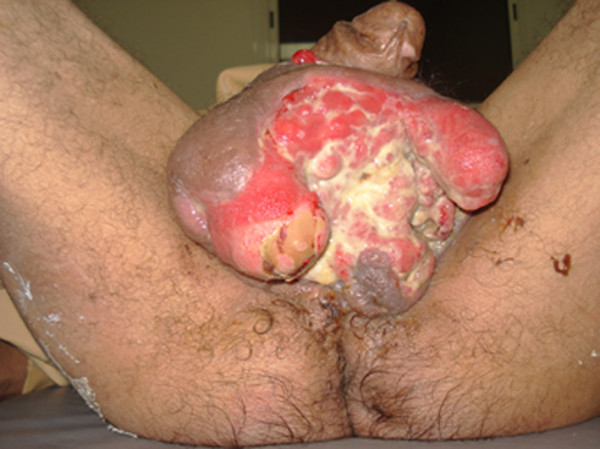
**A mass embracing the perineum, the proximal part of the penis and the upper part of the scrotum**.

**Figure 2:**
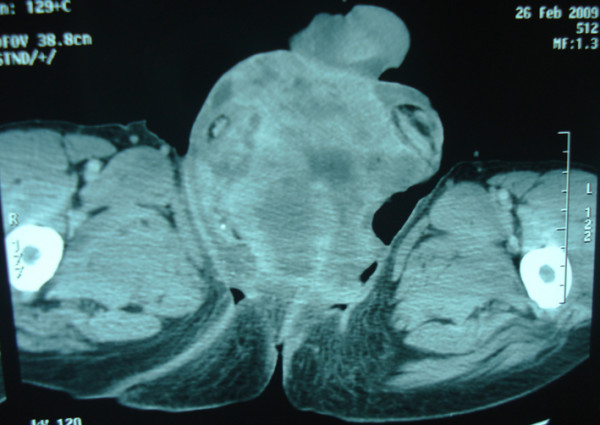
MRI showing a heterogeneous mass arising from the bulbomembranous urethra and invading the proximal part of the cavernous and spongious bodies

**Figure 3:**
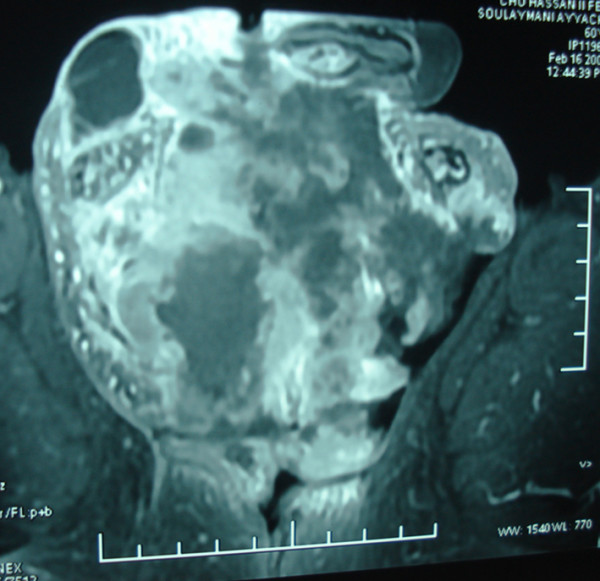
MRI showing that the mass was greatly enhanced, with a clear portion in the nearby tissues.MRI showing that the mass was greatly enhanced, with a clear portion in the nearby tissues

**Figure 4:**
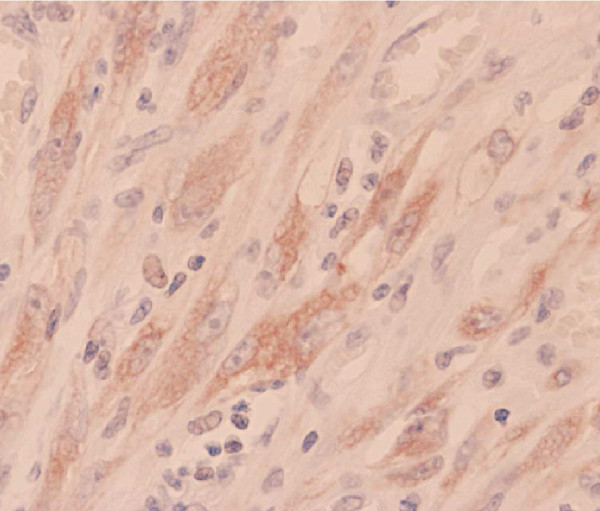
**Proliferation of spindle-shaped cells (HES × 40)**.

**Figure 5:**
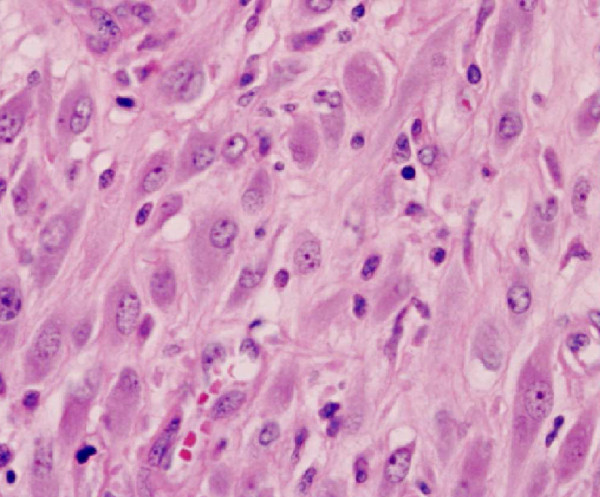
**Immunoreactivity for smooth and common muscle actin (HES × 10)**.

A 65-year-old Moroccan male patient had undergone a perineal urethrostomy to treat an extended distal urethral stricture. After one year, he presented with 3-month history of dysuria, pollakiuria and urethral bleeding. About six weeks previous to examination, slight terminal hematuria was noticed. His weight was stable and he appeared well nourished. Physical examination of the genitals revealed a firm and non-fluctuant mass embracing the perineum, the proximal part of the penis and the upper part of the scrotum (Figure 1). The prostate was small and showed no evidence of nodulation. A magnetic resonance imaging test performed on the patient revealed a 6 cm size heterogeneous mass arising from the bulbomembranous urethra and invading the proximal part of the cavernous and spongious bodies (Figure 2). After injection of contrast gadolinium, the mass was greatly enhanced, with a clear portion in the nearby tissues (Figure 3). A biopsy of the mass was performed, and pathological examination identified characteristics of leiomyosarcoma. Chest roentgenogram, bone scan, liver-spleen scan, and computed tomographic scan of the pelvis were negative for metastatic disease. The patient was surgically treated by pelvic exenteration. Pathological examination showed that the cancer was composed of fascicles of interlacing, moderately large, spindle-shaped cells, with abundant eosinophilic cytoplasm. Immunohistochemistry showed strong staining for smooth muscle actin (SMA) (Figure 4 and Figure 5). The cutting margins were positive. The patient underwent therefore radiotherapy and two cycles of adjuvant chemotherapy consisting of mesna, doxorubicin, ifosfamide and dacarbazine (MAID regimen). However, he developed a local recurrence 4 months after surgery, and the recurrent tumor quickly extended to the pelvic wall. Then, the tumor metastasized to the lungs. The patient died of the disease 7 months after surgery.

## Discussion

Leiomyosarcoma is a rare soft-tissue tumor that accounts for 10-20% of soft-tissue sarcoma. It is generally seen in middle-aged patients, and affects women more frequently than men [1]. Its usual locations, in order of decreasing frequency are: retroperitoneal, intra-abdominal, cutaneous and subcutaneous. Leiomyosarcoma is a highly malignant tumor with an extremely poor prognosis. The 5-year overall survival rate in patients with soft-tissue sarcoma of all stages remains poor, at only 50-60%, and 5-year disease-free survival is rare. To our knowledge, only 1 case of urethral leiomyosarcoma has been previously reported. Wide local excision is considered to be the treatment of choice with radiation and chemotherapy being offered to patients with positive margins or nodes, or those with bulky disease [2,3]. If anatomically feasible, radiotherapy can be considered for larger tumors and/or positive margins. Adjuvant chemotherapy has not been proven to be effective and remains investigational.

## Competing interests

The authors declare that they have no competing interests.

## Authors' contributions

YA analyzed and interpreted the patient data regarding the retroperitoneal disease. MT and ET have made contributions to conception and design, and acquisition of data. AB performed the histological examination of the kidney, and was a major contributor in writing the manuscript. ME have been involved in drafting the manuscript and revising it critically for important intellectual content. MF have given final approval of the version to be published. All authors read and approved the final manuscript.

## Consent

Written informed consent was obtained from the patient for publication of this case report and accompanying images. A copy of the written consent is available for review by the Editor-in-Chief of this journal.
